# Mechanism of PAVA-induced toxicity and inflammation in a cocultured skin cell model

**DOI:** 10.3389/fphar.2025.1531459

**Published:** 2025-02-18

**Authors:** Yunyang Song, Wenjie Cheng, Zhen Wang, Tianqi Zhou, Fanghui Wu, Yifeng Yin, Dan Xu, Yanli Liu

**Affiliations:** ^1^ State Key Laboratory of NBC Protection for Civilian, Beijing, China; ^2^ National Pathogen Collection Center for Aquatic Animals, Shanghai Ocean University, Shanghai, China; ^3^ National Demonstration Center for Experimental Fisheries Science Education, Shanghai Ocean University, Shanghai, China; ^4^ Key Laboratory of Freshwater Aquatic Genetic Resources, Ministry of Agriculture, Shanghai Ocean University, Shanghai, China

**Keywords:** PAVA, cocultured skin cell model, cytotoxic, oxidative stress, apoptosis

## Abstract

**Background:**

Pelargonic acid vanillyl amide (PAVA), a stable synthetic analog of capsaicin, exhibits potential for therapeutic applications; however, it may present cytotoxic and pro-inflammatory risks. This study aims to investigate the injury effects of PAVA on a cocultured skin cell model *in vitro*.

**Methods:**

Human keratinocytes and dermal fibroblasts were co-cultured and exposed to PAVA at concentrations ranging from 12.5 to 200 µM for durations of 5, 24, and 48 h. Cell proliferation was quantified using MTS assays. Morphological changes were observed through microscopy, reactive oxygen species (ROS) production was evaluated via fluorescence analysis, apoptosis was assessed using flow cytometry and Western blotting techniques, while inflammatory cytokines (IL-6, IL-8) were quantified by ELISA.

**Results:**

The proliferation of cells was significantly inhibited by PAVA in a dose- and time-dependent manner, with concentrations of 100 µM and above inducing substantiazl cytotoxicity. Morphological analysis revealed an increase in cell dispersion, irregular morphology, and apoptosis, particularly after prolonged exposure. Treatment with PAVA led to elevated levels of ROS, indicating the presence of oxidative stress. Apoptosis was initiated through both extrinsic pathways (NF-κB, Caspase-8) at an early stage and intrinsic pathways (Caspase-3/9, Bax) at a later period. Furthermore, PAVA markedly increased the secretion of IL-6 and IL-8, suggesting a robust pro-inflammatory response.

**Conclusion:**

100 μM PAVA elicits pronounced cytotoxic, oxidative, and pro-inflammatory effects on cocultured skin cell model, particularly at higher concentrations and prolonged exposure durations. These findings underscore the necessity of exercising caution when employing PAVA for therapeutic purposes and highlight the imperative for further research to mitigate its adverse consequences as a riot control agent.

## 1 Introduction

Capsaicin, the principal bioactive component in chili peppers, is a highly selective agonist of the ion channel protein Transient Receptor Potential Vanilloid 1 (TRPV1). It belongs to the amide-type alkaloid class and more than 30 homologues of capsaicin (Mazourek et al., 2009; Bosland and Votava, 2012) have been identified, all sharing a common core structure. Pelargonic acid vanillyl amide (PAVA), a synthetic analog of capsaicin also known as PAVA (Constant et al., 1996), retains some of its bioactivity while exhibiting superior chemical stability and biocompatibility compared to natural capsaicin. These properties position PAVA as a promising candidate for applications in biomedical research and as a non-lethal agent in crowd control formulations (Yeung and Tang, 2015; Nemala and Nandagopal, 2024).

Oleoresin capsicum (OC) and PAVA act through TRPV1 receptor activation, leading to severe ocular pain, lacrimation, cutaneous burning, and respiratory distress, thereby rapidly incapacitating targeted individuals (Copeland and Nugent, 2013; Yeung and Tang, 2015; Li H. et al., 2023). Consequently, these compounds are widely utilized by law enforcement as essential components of non-lethal tear gas formulations (Huang et al., 2024). Although these compounds are effective for crowd control, the residual PAVA at the task site is challenging to remove effectively through water and existing civilian disinfectants, especially in enclosed spaces, leading to secondary irritation to unprotected individuals. Consequently, their use has raised significant health and safety concerns (Krishnatreyya et al., 2018). On the other hand, the therapeutic potential of capsaicinoids for dermatological conditions has attracted significant attention (Basharat et al., 2021; Mondal et al., 2024). For instance, numerous studies have substantiated their mechanisms in alleviating chronic pruritus and neuropathic pain (Ständer et al., 2010; Anand and Bley, 2011). However, despite their therapeutic applications, the acute pain, irritation, and potential long-term side effects—such as an increased risk when combined with carcinogens—continue to limit the widespread use of capsaicin analogs (Bode and Dong, 2011; Li J. et al., 2023).

As a synthetic analog, PAVA offers the potential to overcome some of these limitations, but its cytotoxicity profile, particularly in the context of skin exposure, when used as a riot control agent or for dermatological treatments, remains underexplored. The skin, being the largest organ of the human body, not only serves as a protective barrier against external physical, chemical, and biological factors but also plays crucial roles in temperature regulation, sensation, and immune defense (Yang et al., 2022). Various chemical products have the potential to elicit local skin reactions upon contact, such as redness and swelling. Therefore, preclinical safety assessments are imperative prior to human exposure to these chemicals, with skin irritation testing emerging as an integral component (Ahn et al., 2010). Initially skin irritation testing relied heavily on animal testing due to the relatively low cost and availability of laboratory animals. However, increasing awareness of animal protection and the development of the 3R principles (Replacement, Reduction, and Refinement) have raised ethical concerns regarding the use of experimental animals. Furthermore, skin irritation tests conducted on animals often result in pain and discomfort, leading to predictive outcomes that do not always correlate with human responses (Welss et al., 2004). In response to these concerns, the 1990s saw the extensive development of coculture models combining keratinocytes and fibroblasts (Gay et al., 1992; Contard et al., 1993; Fleischmajer et al., 1993). The model capitalizes on the naturally occurring crosstalk between keratinocytes and fibroblasts (epidermal-dermal crosstalk), thereby more effectively simulating the physiological environment of human skin (Tan et al., 2017; Jevtic et al., 2020), which makes it an ideal model for assessing skin irritation.

In this study, we established a coculture model of human primary keratinocytes (HSK) and dermal fibroblasts (HSF) to evaluate the effects of PAVA, a TRPV1 receptor agonist. This model allowed us to investigate the comprehensive effects of PAVA, including its impact on cell proliferation, morphology, oxidative stress, apoptosis, signaling pathways, and inflammatory responses, under varying concentrations of PAVA and exposure durations. These findings represent an important step toward understanding the potential adverse effects of PAVA on normal skin tissues and lay the groundwork for future investigations into its safety and applicability in riot control agents and dermatology.

## 2 Materials and methods

### 2.1 Reagents

DMEM/F12 and K-SFM media, 1% (v/v) penicillin-streptomycin, 10% (v/v) fetal bovine serum (FBS), serum extracts, epidermal growth factor (EGF), and pre-stained protein MW marker 26616 were purchased from Thermo Fisher Scientific (Waltham, MA). Human IL-6 and IL-8 ELISA kits were purchased from Ray Biotech (Norcross, GA). Cell Titer^®^ 96 AQueous One Solution Reagent was purchased from Promega (Madison, WI). Dichlorodihydrofluorescin diacetate (DCFH-DA) was obtained from ABP Biosciences (Rockville, MD). Polyvinylidene fluoride membrane was acquired from Millipore (Billerica, MA). Anti-Caspase 3 (ab13847), anti-Caspase 8 (ab25901), anti-Caspase 9 (ab286147), anti-Bax (ab32503), anti-Bad (ab32445), anti-Bcl-2 (ab32124), anti-NF-κB-p65 (ab16502), PI3K/AKT signalling pathway panel (ab283852) and anti-β-actin (ab8227) were purchased from Abcam (Cambridge, United Kingdom). PE Annexin V Apoptosis Detection Kit I was obtained from BD Biosciences (San Diego, CA). IRDye 800 conjugated with affinity purified anti-Rabbit IgG antibody was purchased from Rockland (Philadelphia, PA).

### 2.2 Cell culture

The human primary keratinocytes (HSK) and dermal fibroblasts (HSF) were purchased from National Intrastructure of Cell Line Resource (NICR) in Beijing, China. Human primary dermal fibroblasts were cultured as a monolayer in DMEM/F12 medium supplemented with 1% (v/v) penicillin-streptomycin (100 μg/mL penicillin, 100 μg/mL streptomycin), and 10% (v/v) FBS. Human primary keratinocytes were cultured in K-SFM medium supplemented with 1% (v/v) penicillin-streptomycin, 500 μg/mL serum extracts, and 5 ng/mL EGF. Cells were grown in tissue culture flasks of size 25 cm^2^ at 37°C under an atmosphere containing 5% CO_2_ in a humidified incubator and passaged every 5–7 days.

### 2.3 Construction of a cocultured skin cell model

The cocultured skin cell model was constructed as follows: Human keratinocytes were inoculated at a density of 5 × 10^4^ cells/well in a 6-well plate and cultured at 37°C under an atmosphere of 5% CO_2_ in a humidified incubator. After the keratinocytes adhered, 1.25 × 10^4^ dermal fibroblasts per well were subsequently seeded into the same wells. Similarly, human keratinocytes were seeded at a density of 2 × 10^3^ cells/well in 96-well plates. After cell adhesion, 0.5 × 10^3^ dermal fibroblasts were seeded into the same wells. Cell culture was performed using a mixed medium of K-SFM/DMEM/F12 at a ratio of 3:1:1.

### 2.4 Cell proliferation assay

The proliferation of cocultured skin cell model influenced by PAVA was assessed using the 3-(4, 5-dimethylthiazol-2-yl)-5-(3-carboxy-methoxyphenyl)-2-(4-sulphophenyl)-2H-tetrazolium (MTS) assay. After coculturing in a 96-well plate for 24 h, the cells were washed with PBS (0.01 M, pH 7.4) to remove unattached cells and treated with various concentrations of PAVA (12.5 μM, 50 μM, 100 μM, and 200 μM) for different durations (5 h, 24 h, and 48 h). Subsequently, Cell Titer^®^ 96 AQueous One Solution Reagent (20 μL) was added to each well at at time points of interest (0 h, 5 h, 24 h, and 48 h), followed by incubation for an additional hour. The absorbance at an optical density of 490 nm was measured using a multi-detection microplate reader (BIO-TEK Instruments Inc., Winooski, VT). Mean values and standard errors of mean (SEM) were calculated for each concentration. Cell proliferation and inhibition rates were determined using the formula “Cell proliferation = ${\left({OD}_{PAVA}-{OD}_{Blank}\right)/\left({OD}_{Control}-{OD}_{Blank}\right)}^{\prime \prime }$ and “Cell inhibition = ( 1−Cell proliferation ) × 100%”. The half maximal inhibitory concentration (IC_50_) was analyzed using GraphPad Prism software version 8.0.1.

### 2.5 Fluorescent detection of intracellular ROS

The intracellular level of reactive oxygen species (ROS) in cocultured skin cell model stimulated by PAVA was detected using the dichlorofluorescein (DCF) method. After being cocultured in a 6-well plate for 24 h, the cells were treated with PAVA at concentrations of 100 μM for durations of 5 h and 24 h. Subsequently, they were washed twice with PBS (0.01 M, pH7.4), incubated with 15 μM DCFH-DA for 30 min at 37°C, and then washed three times with HBSS (Thermo Fisher Scientific, United States) before being incubated with DMFM/F12 medium at 37°C for 10 min. Finally, the fluorescent signal was measured using a Cytation cell imaging multi-mode reader (BIO-TEK Instruments Inc., Winooski, VT) with an excitation wavelength set to 485 nm and an emission wavelength set to 590 nm.

### 2.6 Flow cytometry

The type of cell death was investigated using flow cytometry. HSK and HSF were co-cultured in a 6-well plate and treated with PAVA for 5 h and 24 h. After collection and rinsing with PBS (0.01 M, pH 7.4), the cells were resuspended with 100 μL binding buffer (10 mM HEPES pH 7.4, 140 mM NaCl, and 2.5 mM CaCl_2_) containing 5 μL 7-ADD and 5 μL Annexin V-PE, followed by incubation in the dark at room temperature for 15 min. Subsequently, 400 μL of binding buffer was added to the sample tubes, which were then analyzed using FACS Calibur (Becton Dicknson, Franklin Lakes, NJ). At least 10,000 events were measured for each sample in both the FL1 channel (orange-red fluorescence: 575/25 nm) and the FL2 channel (far-red fluorescence: 670/30 nm). Data analysis was performed using FlowJo™ software (version 10.0.6, Tree Star, Ashland, OR).

### 2.7 Western blot analysis

The apoptosis-related proteins were assayed using Western blot according to the established protocol. Briefly, cocultured cells treated with 100 μM PAVA for 5 h, 24 h, and 48 h were harvested. Subsequently, the proteins were resolved on a 15% SDS-PAGE gel and transferred onto polyvinylidene fluoride membranes. These membranes were then incubated overnight at 4°C with polyclonal rabbit antibodies against anti-Caspase 3, anti-Caspase 8, anti-Caspase 9, anti-NF-κB-p65, anti-Bax, anti-Bad, anti-Bcl-2 and anti-β-actin (Abcam, Cambridge, United Kingdom). Afterward, the membranes were incubated with IRDye800-conjugated affinity purified anti-Rabbit IgG antibody for 1.5 h at room temperature. Finally, the membranes were scanned using the Odyssey Infrared Imaging System (LI-COR Bioscience, Lincoln, NE).

### 2.8 ELISA assay of cytokine IL-6 and IL-8

The secretion levels of interleukins (IL-6 and IL-8) from cocultured skin cell model were measured using the enzyme linked immunosorbent assay (ELISA). Following the standard operating procedures of Human IL-6 and IL-8 ELISA kit, supernatants from 4 to 5 × 10^5^ cocultured skin cell model treated with 100 μM PAVA for 5 h, 24 h and 48 h were collected for ELISA analysis to determine the secretion of IL-6 and IL-8. Absorbance was measured at an optical density of 450 nm using the multi-detection microplate reader (BIO-TEK Instruments Inc., Winooski, VT).

### 2.9 Statistical analysis

Statistical analysis was conducted using GraphPad Prism 8.0.1 software. Differences between groups were analyzed using *One-Way ANOVA* followed by *Dunnett’s post hoc test* for multiple comparisons to compare each treatment group with the control group. For comparisons between two groups, an *unpaired t-test* was used. All *in vitro* experiments were performed in triplicate and repeated three times, with the results presented as the Mean ± SEM. *P* value <0.05 is considered statistically significant. *P <0.05; **P <0.01; ***P <0.001; ****P <0.0001.

## 3 Results

### 3.1 Inhibitory effect of PAVA on cell proliferation in cocultured skin cell model

**FIGURE 1:**
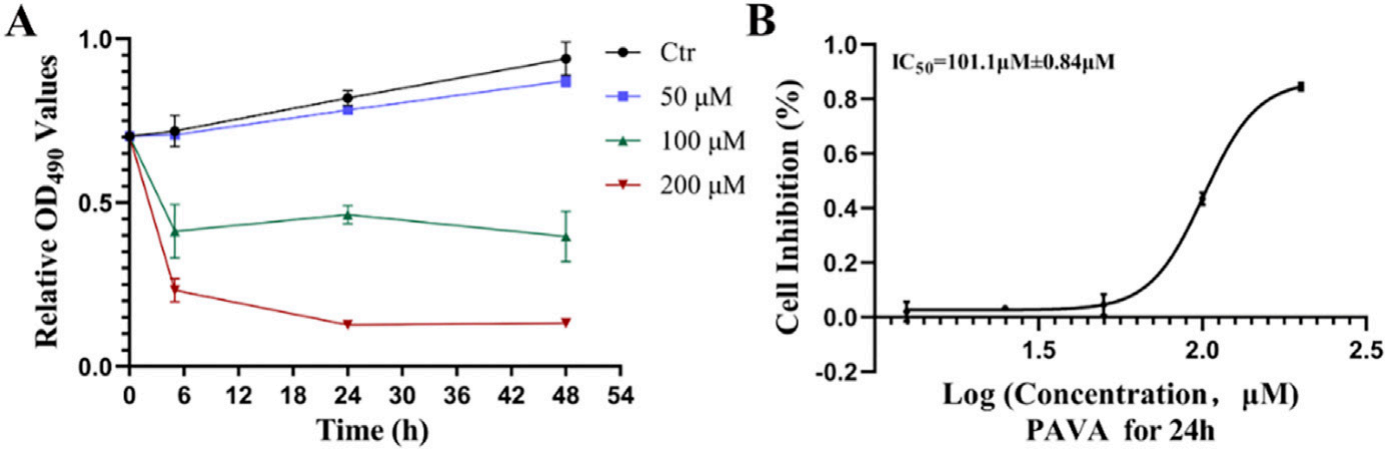
Effects and inhibition curve of PAVA on cell proliferation in cocultured skin cell model. The proliferation of cells treated with PAVA was analyzed by MTS assay at 0 h, 5 h, 24 h and 48 h. **(A)** Effect of PAVA on the proliferation of cocultured skin cell model in dose of 50 μM, 100 μM and 200 μM; **(B)** Cytotoxicity was represented as the concentration of PAVA inhibiting cell growth at 24 h by 50% (IC_50_).

To investigate the impact of PAVA treatment on cell proliferation in a constructed cocultured skin cell model, this study applied different concentrations of PAVA (50 μM, 100 μM, and 200 μM) to this model for varying durations (5h, 24h, and 48 h). While no significant effect on cell proliferation was observed with 50 μM PAVA compared to the control group, both 100 μM and 200 μM PAVA exhibited varying degrees of impact on cell proliferation, with the most pronounced effect observed at a concentration of 200 μM (Figure 1A). The MTS assay results (Figure 1A) demonstrated that a dose-dependent inhibitory effect on cell proliferation in the cocultured skin cell model after a treatment duration of 24 h with different concentrations of PAVA ranging from 12.5 μM to 200 μM. Compared to the control group, inhibition rates ranged from 0.021% ± 0.036% (12.5 μM) to 0.85% ± 0.014% (200 μM) for cocultured skin cell model which were treated with various concentrations of PAVA between 12.5–200 μM (Figure 1B). Furthermore, the IC_50_ of PAVA against cocultured skin cell model was determined to be 101.1 μM ± 0.84 μM (approximately 29.6 mg/L).

### 3.2 Effects of PAVA on cell morphology in cocultured skin cell model

**FIGURE 2:**
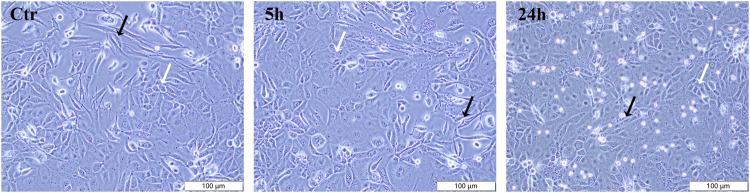
Effects of PAVA on cell morphology in cocultured skin cell model. Cocultured cells treated with PAVA were observed and photographed by microscopy, in ×10 magnifications. Scale bar was 100 μm. Control group cells and cells treated with PAVA for 5 h and 24 h were shown, respectively. White arrows indicate human primary keratinocytes (HSK), and black arrows indicate dermal fibroblasts (HSF).

The morphological impact of PAVA (100 μM) on cells in cocultured skin cell model was observed using inverted microscopy. In the control group, both cell types exhibited morphology consistent with their characteristic features (fibroblasts: long spindle-shaped, full and stretched cells with clear cell outline; keratinocytes: cobblestone-like appearance with defined borders), with a uniform distribution and clear cell boundaries, indicating a healthy state (Figure 2). Treatment with 100 μM PAVA for 5 h induced morphological changes, including slight cell swelling, less distinct cell borders, and minimal alterations in intercellular spacing, although the overall integrity of the cells remained essentially unchanged. After 24 h of treatment with 100 μM PAVA, more pronounced morphological changes were observed, including irregular cell shapes, loss of clear cell contours in many cells, a reduction in cell size with rounding and loss of characteristic features in numerous cells, and clear signs of apoptosis (Figure 2).

### 3.3 Effects of PAVA on oxidative stress in cocultured skin cell model

**FIGURE 3:**
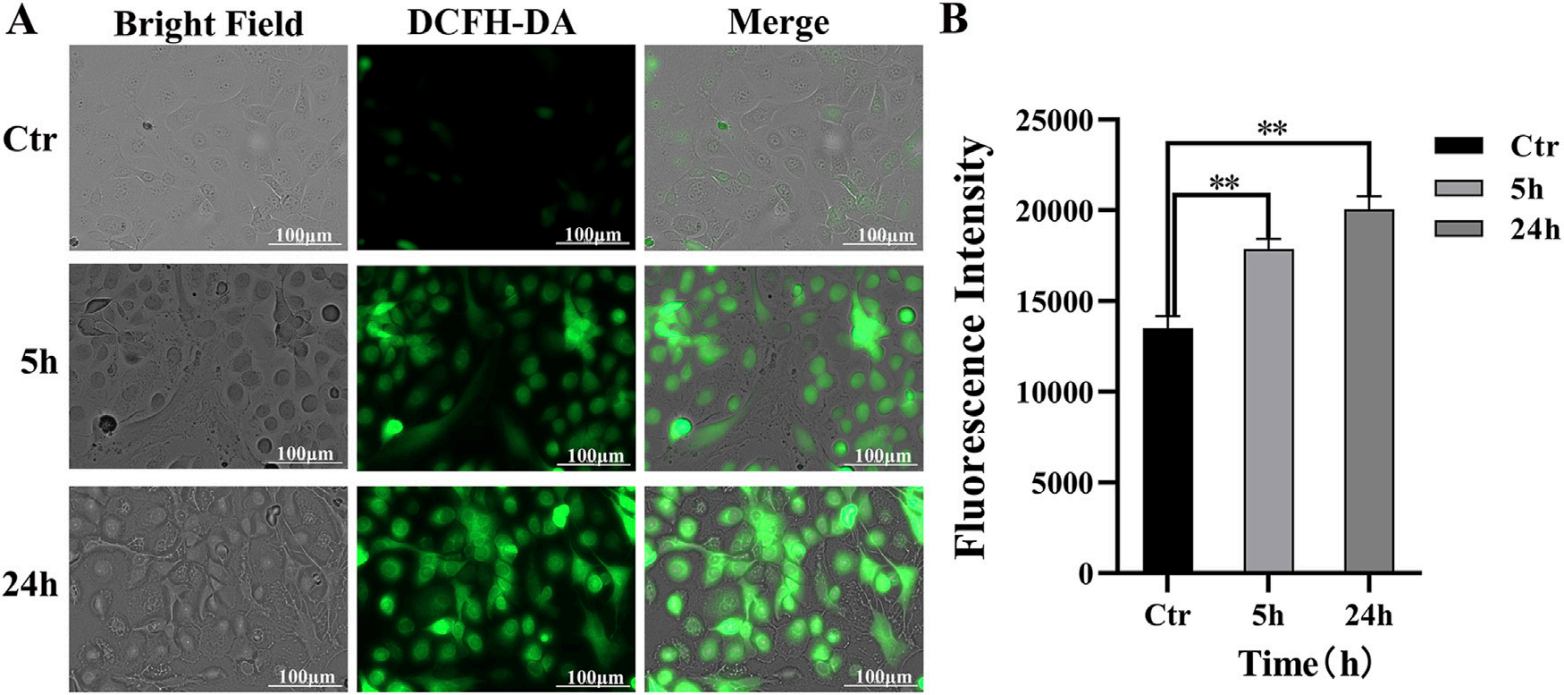
Intracellular ROS response induced by PAVA in cocultured skin cell model. The cocultured skin cell model were pretreated with 100 μM PAVA for 5 h and 24 h, and the production of ROS was detected by cell fluorescence imaging system using DCFH-DA. **(A)** The treatment with PAVA for 5 h and 24 h led to accumulation of cellular ROS (green color) compared with the control condition (black and white color), in ×20 magnifications. Scale bar was 100 μm; **(B)** The relative fluorescence intensity of cocultured skin cell model.

The generation of ROS in cocultured skin cell model was evaluated using fluorescence microscopy under various conditions (Figure 3A). Minimal green fluorescence was observed in the control group exhibited, indicating low levels of ROS and negligible oxidative stress. Treatment with 100 μM PAVA for 5 h resulted in an increase in green fluorescence spots, suggesting that PAVA treatment induced oxidative stress and enhanced intracellular ROS generation. A low-magnification view (4×) of the visual field is provided in supplementary materials Supplementary Figure S1 to further illustrate the observed effects. After 24 h of exposure to the same concentration of PAVA, the intensity of green fluorescence significantly intensified compared to the 5-h treatment group, demonstrating cumulative effects of oxidative stress over time (Figure 3B).

### 3.4 Effects of PAVA on apoptosis in cocultured skin cell model

**FIGURE 4:**
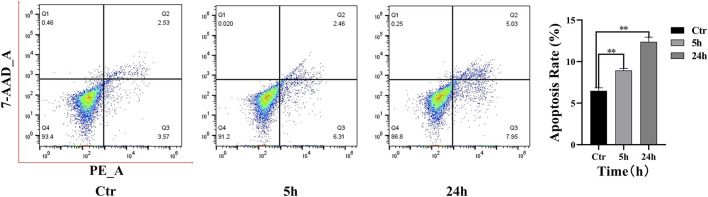
Flow cytometry analysis of PAVA-stimulated cocultured skin cell model. Flow cytometric assay was performed by binding of Annexin V-PE and uptake of 7-ADD to evaluate the early apoptosis and late apoptosis. The histogram was represented as the Mean ± SEM of three independent experiments. Q1, dead cells (7-ADD-positive, annexin V-PE-negative), Q2, late apoptotic cells (7-ADD-positive, annexin V-PE-positive); Q3, early apoptotic cells (7-ADD-negative, annexin V-PE-positive); Q4, living cells (7-ADD-negative, annexin V-PE-negative); Values in the Q2 and Q3 quadrants represent percentage of cells showing early apoptotic and late apoptotic, respectively. **P < 0.01 versus control.

The level of apoptosis in cells following PAVA treatment was quantitatively analyzed using flow cytometry results (Figure 4). In the control group, minimal apoptosis was observed, with the majority of cells (93.40%) located in the live cell quadrant (Q4). After treatment with 100 µM PAVA for 5 h, a slight increase in early apoptotic cells (Q3: 6.31%) was observed. By 24 h, both early apoptotic cells (Q3: 7.95%) and late apoptotic cells (Q2: 5.03%) exhibited an increase. Compared to the control group, the overall apoptotic rate in cocultured skin cell model treated with PAVA for 5 and 24 h was notably elevated, indicating that the apoptotic effects accumulate over time. Specific cell gating strategies are provided in the supplementary materials (Supplementary Figure S2).

### 3.5 Effects of PAVA on cell signaling pathways in cocultured skin cell model

**FIGURE 5:**
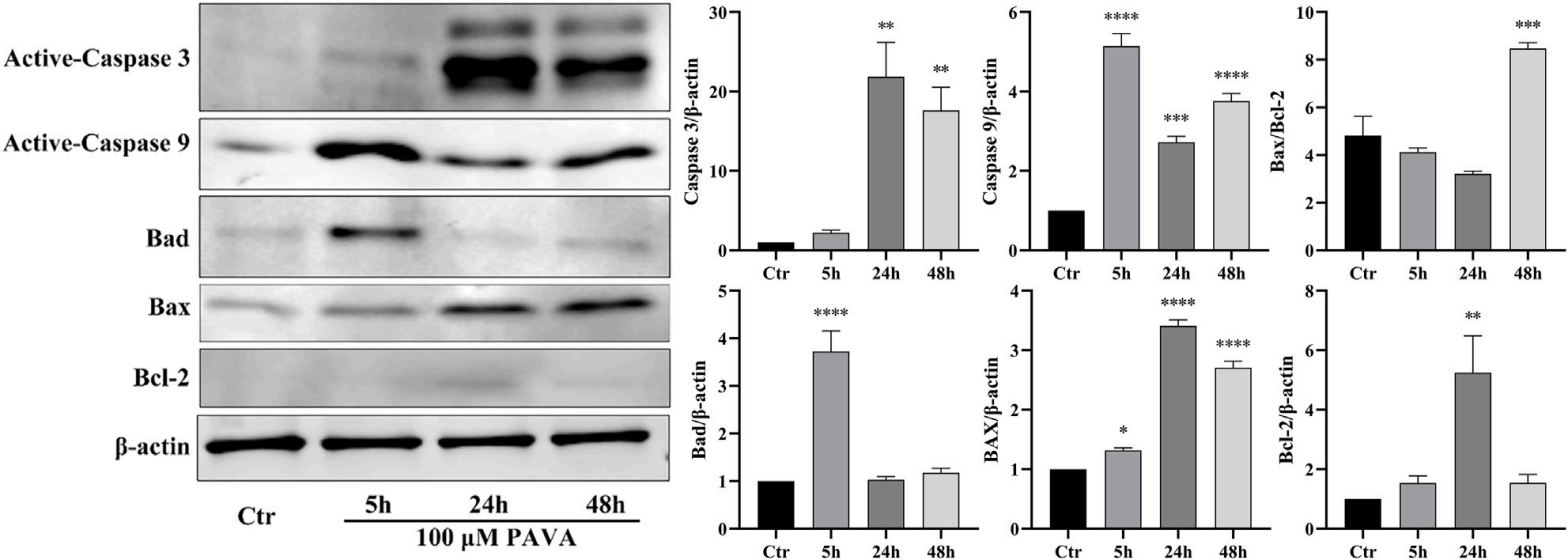
Changes in Caspase-3/9, Bad, Bax, and Bcl-2 expression levels in PAVA-stimulated cocultured skin cell model. After treated with 100 μM PAVA for 5 h, 24 h, and 48 h, cocultured skin cell model were harvested and lysed. Whole proteins were separated on SDS-PAGE gel and then subjected to immunoblot analysis with anti-Caspase 3, anti-Caspase 9, anti-Bax, anti-Bad, anti-Band anti-β-actin antibody on the same membrane, followed by IRDye 800 conjugated with affinity purified anti-Rabbit IgG secondary antibody. β-actin was used as a loading control.

**FIGURE 6:**
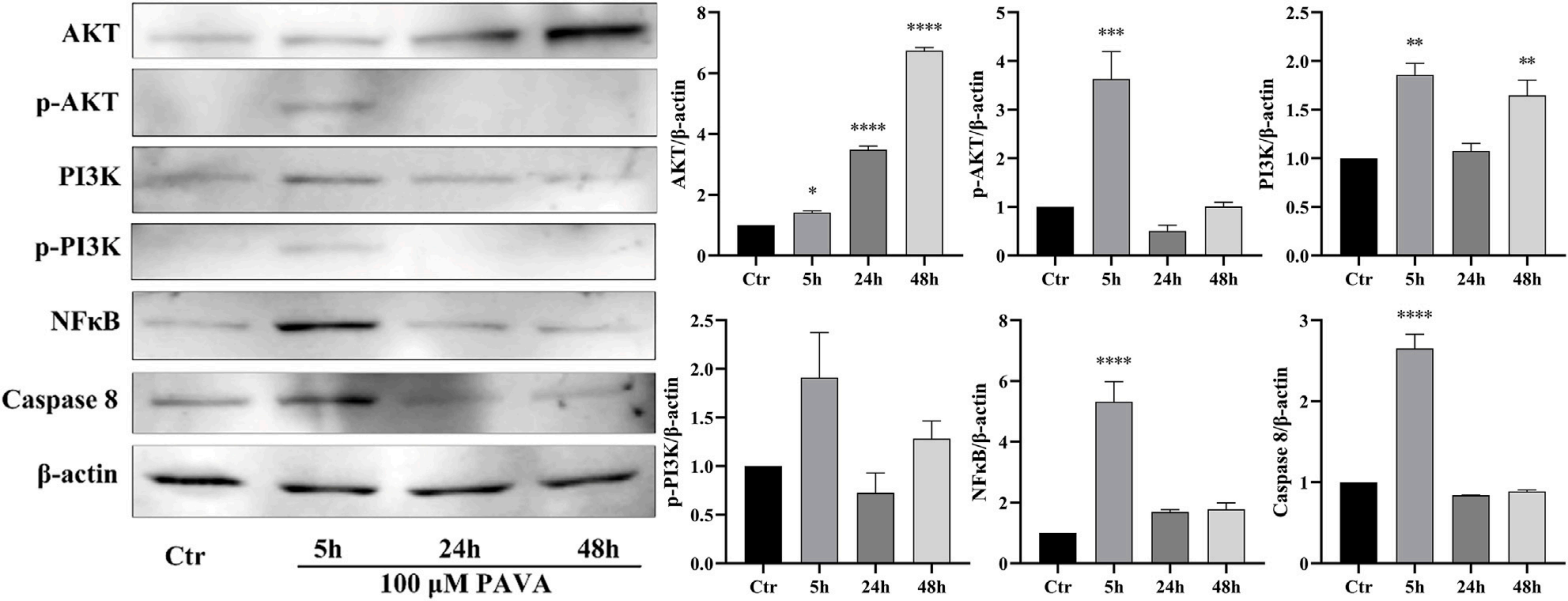
Changes in PI3K/AKT, NF-κB, and Caspase-8 expression levels in PAVA-stimulated cocultured skin cell model. After treated with 100 μΜ PAVA for 5 h, 24 h, and 48 h, cocultured skin cell model were harvested and lysed. Whole proteins were separated on SDS-PAGE gel and then subjected to immunoblot analysis with anti-PI3K/AKT, anti- NF-κB, anti-Caspase 8, anti-NFκB, and anti-β-actin antibody on the same membrane, followed by IRDye 800 conjugated with affinity purified anti-Rabbit IgG secondary antibody. β-actin was used as a loading control.

Cell apoptosis is a complex process involving intricate interplay among multiple pathways. Combining results from oxidative stress and flow cytometry analysis, this study employed Western blot protein immunoblotting to examine apoptosis-related protein molecules. The results revealed significant upregulation of activated Caspase-3 and Caspase-9 expression levels at 5 h, 24 h, and 48 h following treatment with 100 μM PAVA. Moreover, an increase in the levels of pro-apoptotic proteins Bad and Bax indicated activation of the mitochondrial pathway of apoptosis (Figure 5). With prolonged exposure to PAVA, the expression of Bad predominantly decreased; however, the expression of pro-apoptotic protein Bax remained significantly elevated along with a relative increase in the Bax/Bcl-2 ratio. Furthermore, after 5 h treatment with 100 μM PAVA, there was a notable increase in Caspase-8 expression levels (Figure 6), suggesting that PAVA may also induce apoptosis through the Caspase-8-dependent pathway, another crucial apoptotic signaling molecule.

To gain a comprehensive understanding of cellular responses under PAVA stimulation, we also assessed key molecules in the PI3K/AKT signaling pathway and NF-κB signaling pathway, which are crucial for cell survival pathways. The results revealed a significant increase in the expression levels of phosphorylated AKT (p-AKT), phosphorylated PI3K (p-PI3K), and NF-κB after 5 h of treatment with 100 μM PAVA. However, as the duration of treatment increased, the levels of p-AKT, p-PI3K, and NF-κB decreased (Figure 6). These findings suggest that early PAVA treatment (5 h) initiated cell apoptosis and pro-inflammatory responses through activation of apoptotic factors and NF-κB signaling. By 24 h, the mitochondrial pathway became the predominant apoptotic pathway due to upregulation of Caspase-3/9 and Bax. Concurrently, pro-inflammatory responses persisted, although there was a decline in NF-κB expression possibly due to sustained stimulation leading to negative feedback regulation. In the late stage of PAVA treatment (48 h), apoptotic signals persisted, with cell apoptosis becoming the dominant response. Meanwhile, NF-κB signaling further decreased, indicating an impaired ability of cells to sustain survival mechanisms.

### 3.6 Pro-inflammatory effects of PAVA on cells in cocultured skin cell model

**FIGURE 7:**
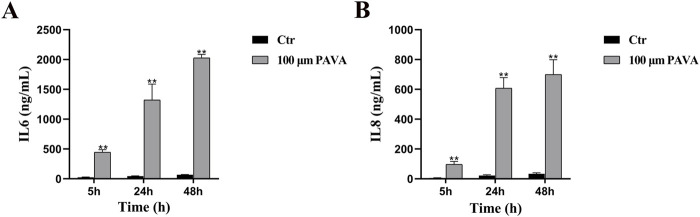
Effect of PAVA on IL-6 and IL-8 secretion in cocultured skin cell model. The supernatants from the cocultured skin cell model treated with 100 μM PAVA for 5 h, 24 h, and 48 h were collected for IL-6 **(A)** and IL-8 **(B)** ELISA. Data represent the mean of OD450 ± SEM of three independent experiments. **P < 0.01 versus control.

Given the pronounced upregulation of NF-κB observed in early stages of PAVA treatment, it is postulated that PAVA-induced cellular apoptosis may ultimately be associated with sustained inflammatory responses. In this study, sandwich ELISA was employed to detect secreted pro-inflammatory cytokines (IL-6 and IL-8). The results revealed a significant upregulation in the secretion of IL-6 (Figure 7A) and IL-8 (Figure 7B) following PAVA treatment, suggesting the potential capacity of PAVA to stimulate the production and release of pro-inflammatory cytokines by cells.

Considering all experimental findings, it is evident that the effects of PAVA treatment on cells exhibit a time-dependent nature. Initially, PAVA triggers apoptotic signals and pro-inflammatory responses through multiple pathways. Subsequently, the mitochondrial pathway gradually emerges as the predominant apoptotic pathway, while survival signaling pathways are progressively attenuated.

## 4 Discussion

This study presents the first evaluation of PAVA’s effects on a cocultured skin cell model, highlighting its dose- and time-dependent impacts on cell proliferation, morphology, oxidative stress, apoptosis, signaling pathways, and inflammatory responses. The findings demonstrate that PAVA significantly inhibits cell proliferation in a dose- and time-dependent manner, with 200 μM exerting a more pronounced suppressive effect than 100 μM. Morphological analysis shows that PAVA treatment induces cell swelling, blurred boundaries, and irregular shapes, which may indicate early signs of cellular stress and apoptosis (Bottone et al., 2013). After 24 h of exposure, these morphological alterations became more pronounced, exhibiting clear apoptotic features, which further support the hypothesis that PAVA induces apoptosis in the cocultured skin cell model.

Oxidative stress, a key trigger of apoptosis (Circu and Aw, 2010), emerged as a central mechanism in PAVA-induced cellular effects. Our study showed that PAVA significantly increased ROS levels in the cocultured skin cell model, with oxidative stress effects intensifying over time. Elevated ROS likely causes direct damage to cellular components, including membrane lipids, proteins, and DNA, thereby triggering intracellular signaling cascades (Circu and Aw, 2010). Excess ROS in mitochondria may destabilize membrane potential, promote cytochrome c release, and activate Caspase-9, initiating the mitochondrial-dependent apoptotic pathway (Chen et al., 2024). Western blot analysis further confirmed these findings, showing significant upregulation of active Caspase-3 and Caspase-9, along with increased levels of pro-apoptotic proteins Bad and Bax. Overall, these results suggest that the mitochondrial pathway is the primary driver of PAVA-induced cell death. Sensitivity to ROS likely varies among different cell types (Hacioglu, 2022). Our findings indicate that ROS levels induced by 100 μM PAVA may surpass the antioxidant capacity of cells in the cocultured skin cell model, leading to oxidative stress and related damage. Supporting this, previous studies have demonstrated that capsaicinoids in other cellular models induce apoptosis via ROS generation (Pramanik et al., 2011; Pawar et al., 2022). One *in vitro* study found that capsaicin induced excessive ROS production in human dermal fibroblasts, triggering inflammatory responses and promoting cell death (Hudita et al., 2021). This aligns with our findings, indicating a conserved mechanism across different models. Despite these insights, the limitations of the coculture model prevent a definitive determination of which cell type primarily drives the observed apoptotic responses. Nevertheless, our findings strongly suggest that PAVA markedly amplifies oxidative stress in the cocultured skin cell model, potentially serving as a key mechanism underlying its pro-apoptotic effects.

Oxidative stress occurs when the body’s antioxidant system fails to effectively eliminate ROS, resulting in skin cell aging, inflammation, and carcinogenesis. This process involves two primary mechanisms: (1) direct oxidative damage to biomolecules by ROS and (2) ROS-mediated modulation of cellular signaling pathways that alter gene expression (Liu et al., 2023). In this study, PAVA treatment for 5 h increased the expression levels of phosphorylated PI3K (p-PI3K), AKT (p-AKT), and NF-κB. The PI3K/AKT/NF-κB signaling pathway is a well-established regulator of skin homeostasis, influencing various processes such as inflammation, cell proliferation, differentiation, apoptosis, angiogenesis, metabolism, and protein synthesis (Yoon et al., 2020; Teng et al., 2021). These findings indicate that in the early stages of PAVA exposure, cocultured skin cell model activate survival signals to counter external stimuli. With prolonged PAVA treatment, the PI3K/AKT and NF-κB pathways were progressively suppressed, suggesting that sustained oxidative stress impaired cellular survival. NF-κB, a central mediator of inflammatory pathways, regulates the transcription of genes encoding pro-inflammatory cytokines, chemokines, and growth factors, all linked to the onset and persistence of skin diseases (Queiro et al., 2021). Previous *in vivo* studies showed that capsaicin enhances the expression of pro-inflammatory markers like COX-2 and iNOS and activates NF-κB, exacerbating inflammation and promoting skin tumorigenesis in a DMBA/TPA-induced model (Liu et al., 2015). Moreover, *in vitro* studies have shown that capsaicin exposure triggers TRPV1-mediated Ca^2+^ influx in keratinocytes, leading to the release of inflammatory cytokines such as IL-6, IL-8, and TNF-α, which are linked to erythema, capillary dilation, and pain (Lee et al., 2009; Lee et al., 2023; Xiao et al., 2023). In our study, PAVA treatment significantly increased the secretion of pro-inflammatory cytokines IL-6 and IL-8 in the cocultured skin cell model, further supporting its role in amplifying inflammatory responses. ELISA results showed sustained upregulation of IL-6 and IL-8 levels, indicating that the NF-κB pathway is critical for regulating inflammation-related gene expression in the early stages of PAVA exposure. Although NF-κB expression decreased in the later stages of treatment, its early activation likely established a pro-inflammatory environment, which facilitated apoptosis through mitochondrial pathways.

In summary, PAVA induces oxidative stress in the cocultured skin cell model by increasing ROS production, which activates the PI3K/AKT/NF-κB signaling pathway to promote cell survival and pro-inflammatory responses. Over time, PI3K/AKT/NF-κB pathway activity decreases, while the mitochondrial apoptotic pathway predominates, ultimately resulting in cellular damage and death. Interestingly, previous studies have demonstrated that soluble factors secreted by keratinocytes are essential for NF-κB activation in fibroblasts, whereas direct exposure to stimuli like sodium dodecyl sulphate (SDS) or potassium diformate (Formi^®^) does not activate NF-κB (Canton et al., 2010). This suggests that paracrine factors secreted by keratinocytes play a critical role in fibroblast activation (Russo et al., 2020; Cooper et al., 2021). Although our study identifies multiple mechanisms of PAVA-induced damage in the cocultured skin cell model, it remains unclear whether these effects are directly caused by PAVA or mediated through paracrine regulation. This limitation hinders a complete understanding of PAVA’s cytotoxic mechanisms. Future studies using contactless growth models, such as Transwell systems, or monoculture models are necessary to clarify the toxicity and signaling pathways associated with PAVA in specific cell types. Overall, this study highlights that PAVA stimulation induces prolonged inflammatory responses in the cocultured skin cell model. Given PAVA’s potential applications as both a riot control agent and a therapeutic agent for dermatological conditions, its use inevitably involves direct skin exposure. These findings underscore the necessity of thoroughly evaluating PAVA’s safety, particularly its cytotoxic and inflammatory effects, to mitigate risks associated with its application.

## Data Availability

The original contributions presented in the study are included in the article/Supplementary Material, further inquiries can be directed to the corresponding authors.
