# Supplementary material for: Mechanism of PAVA-induced toxicity and inflammation in a cocultured skin cell model
Source: Front Pharmacol. 2025 Feb 18;16:1531459. doi: 10.3389/fphar.2025.1531459 (PMC11876130; doi:10.3389/fphar.2025.1531459)
Supplement: Supplementary file 1 [file DataSheet1.pdf]

frontiers

Supplementary Material

Mechanism of PAVA-Induced toxicity and inflammation in A Cocultured Skin Cell Model

Yunyang Song[1#], Wenjie Cheng[2,3,4#], Zhen Wang[1], Tianqi Zhou[2,3,4], Fanghui Wu[1], Yifeng Yin[1], Dan Xu[2,3,4*], Yanli Liu[1*]

[1]State Key Laboratory of NBC Protection for Civilian, Beijing 102205, China

[2]National Pathogen Collection Center for Aquatic Animals, Shanghai Ocean University, Shanghai 201306, China

[3]National Demonstration Center for Experimental Fisheries Science Education, Shanghai Ocean University, Shanghai 201306, China

[4]Key Laboratory of Freshwater Aquatic Genetic Resources, Ministry of Agriculture, Shanghai Ocean University, Shanghai 201306, China

*** Correspondence:**

Corresponding Author: dxu@shou.edu.cn (Dan Xu), liuh3062023@163.com (Yanli Liu).
[#]These authors contributed equally to this work.

1 Supplementary Figures

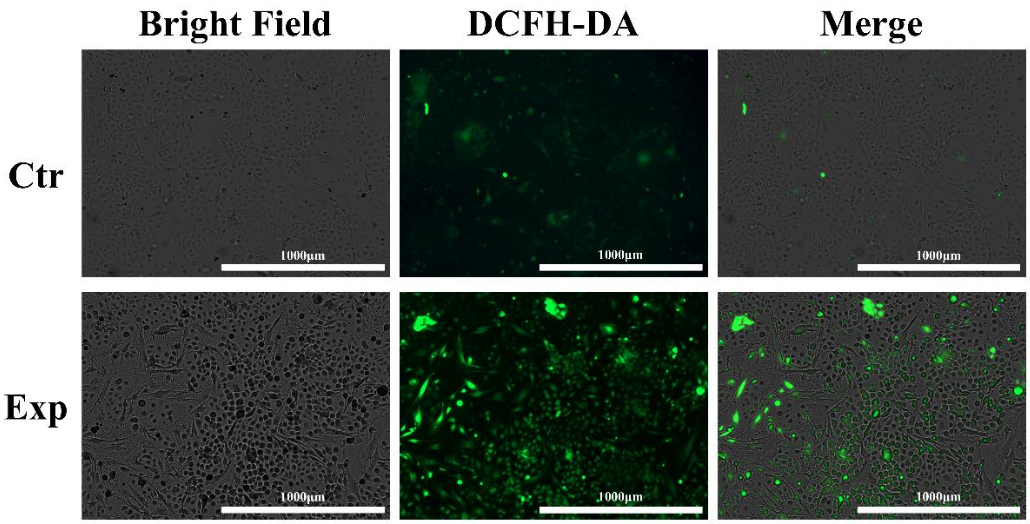

Figure S1. Intracellular ROS response induced by PAVA in cocultured skin cell model. Bright field (left), DCFH-DA staining indicating ROS levels (middle), and merged images (right) are shown for

both control (Ctr, top row) and experimental (Exp, bottom row) conditions. Images were captured at 4× magnification, scale bar was 1000 µm.

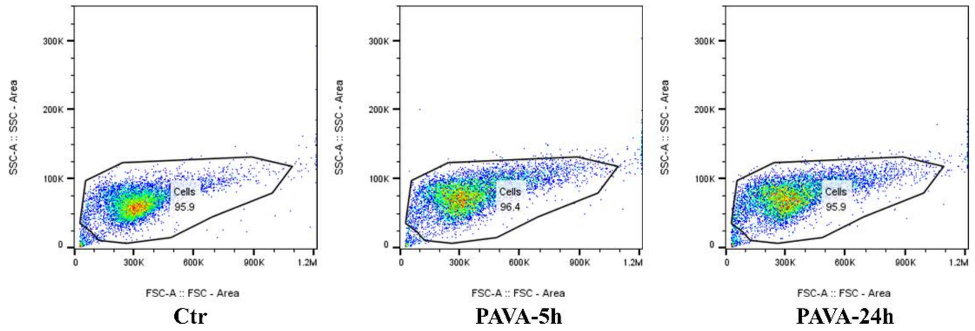

Figure S2. Flow cytometric analysis illustrating the effect of PAVA treatment on cell populations at different time intervals. FSC-A (Forward Scatter Area) and SSC-A (Side Scatter Area) are used to assess cell size and internal complexity, respectively. The 'Cells' region in each plot represents the selected cell population for analysis.
